# (*E*)-Methyl *N*′-(4-hydroxy­benzyl­idene)hydrazinecarboxyl­ate

**DOI:** 10.1107/S1600536808018096

**Published:** 2008-06-19

**Authors:** Xiang-Wei Cheng

**Affiliations:** a Zhejiang Police College Experience Center, Zhejiang Police College, Hangzhou 310053, People’s Republic of China

## Abstract

In the title compound, C_9_H_10_N_2_O_3_, the hydroxy group and the C=N—N unit are coplanar with the benzene ring. The benzene rings of inversion-related mol­ecules are stacked with their centroids separated by a distance of 3.7703 (9) Å, indicating weak π–π inter­actions. In the crystal structure, C—H⋯O, O—H⋯O, N—H⋯O and C—H⋯O hydrogen bonds link molecules into a infinite two-dimensional network along the *a* axis.

## Related literature

For general background, see: Hadjoudis *et al.* (1987 ▶); Borg *et al.* (1999 ▶); Parashar *et al.* (2005). For a related structure, see: Shang *et al.* (2007 ▶). For related literature, see: Parashar *et al.* (1988 ▶).
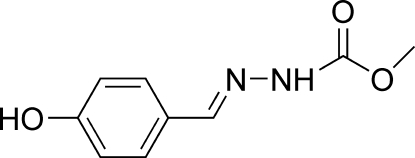


## Experimental

### 

#### Crystal data

C_9_H_10_N_2_O_3_
*M* *_r_* = 194.19
Monoclinic, 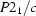

*a* = 8.1943 (8) Å
*b* = 12.0512 (11) Å
*c* = 10.1067 (9) Å
β = 111.970 (3)°
*V* = 925.57 (15) Å^3^
*Z* = 4
Mo *K*α radiation
μ = 0.11 mm^−1^
*T* = 123 (2) K
0.31 × 0.28 × 0.24 mm

#### Data collection

Bruker SMART CCD area-detector diffractometer
Absorption correction: multi-scan (*SADABS*; Bruker, 2002 ▶) *T*_min_ = 0.969, *T*_max_ = 0.978
9552 measured reflections
1623 independent reflections
1487 reflections with *I* > 2σ(*I*)
*R*_int_ = 0.021

#### Refinement

*R*[*F* ^2^ > 2σ(*F* ^2^)] = 0.035
*wR*(*F* ^2^) = 0.115
*S* = 0.95
1623 reflections
128 parameters
H-atom parameters constrained
Δρ_max_ = 0.24 e Å^−3^
Δρ_min_ = −0.19 e Å^−3^

### 

Data collection: *SMART* (Bruker, 2002 ▶); cell refinement: *SAINT* (Bruker, 2002 ▶); data reduction: *SAINT*; program(s) used to solve structure: *SHELXS97* (Sheldrick, 2008 ▶); program(s) used to refine structure: *SHELXL97* (Sheldrick, 2008 ▶); molecular graphics: *SHELXTL* (Sheldrick, 2008 ▶); software used to prepare material for publication: *SHELXTL*.

## Supplementary Material

Crystal structure: contains datablocks I, global. DOI: 10.1107/S1600536808018096/tk2273sup1.cif

Structure factors: contains datablocks I. DOI: 10.1107/S1600536808018096/tk2273Isup2.hkl

Additional supplementary materials:  crystallographic information; 3D view; checkCIF report

## Figures and Tables

**Table 1:** Hydrogen-bond geometry (Å, °)

*D*—H⋯*A*	*D*—H	H⋯*A*	*D*⋯*A*	*D*—H⋯*A*
O1—H1⋯O2^i^	0.84	2.58	3.068 (2)	118
O1—H1⋯N1^i^	0.84	2.11	2.941 (2)	169
N2—H2*A*⋯O2^ii^	0.88	2.13	2.964 (2)	158
C7—H7⋯O2^ii^	0.95	2.38	3.188 (2)	143

Symmetry codes: (i) 
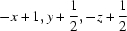
; (ii) 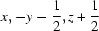.

**Fig. 1.**
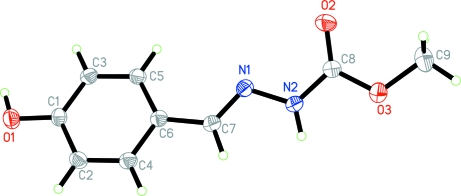
Molecular structure of (I), showing 30% probability displacement ellipsoids and the atomic numbering.

## supplementary crystallographic information

### Comment

Benzaldehydehydrazone derivatives have received considerable attention owing to their pharmacological activity (Parashar *et al.*, 1988) and their photochromic properties (Hadjoudis *et al.*, 1987). In addition, they are important intermediates in the synthesis of 1,3,4-oxadiazoles, which have been reported to be versatile compounds (Borg *et al.*, 1999). As a part of an investigation of this type of derivative, the crystal structure of the title compound, C_9_H_10_N_2_O_3_ (I), is described herein.

In (I), Fig. 1 & Table 1, all non-hydrogen atoms are co-planar to within ±0.699 (4) Å. The molecule is in the E-conformation with respect to the N=C double bond. The bond lengths and angles defining the C=N—N(H)—C group are close to those of the previously reported N'-(4-Methoxybenzylidene)methoxyformohydrazide structure (shang *et al.*, 2007).

The benzene rings of inversion-related molecules are stacked with their centroids separated by a distance of 3.7703 (9) Å, consistent with π-π interactions.

### Experimental

4-Hydroxy benzaldehyde (12.2 g, 0.1 mol) and methyl hydrazinecarboxylate (9.0 g, 0.1 mol) were dissolved in methanol (50 ml) solution and stirred for 6 h at room temperature. The resulting solid was filtered off and recrystallized from an ethanol solution to give (I) in 80% yield. Crystals suitable for X-ray analysis were obtained by the slow evaporation of an ethanol solution held at room temperature (m.p. 475–478 K).

### Refinement

The H atoms were included in the riding model approximation with O—H = 0.84 Å, N—H = 0.86 Å and C—H = 0.95 - 0.98 Å, and with *U*_iso_(H) = 1.2*U*_eq_(C, N) and 1.5*U*_eq_(O, methyl-C).

### Crystal data

**Table:**

C_9_H_10_N_2_O_3_	*F*_000_ = 408
*M**_r_* = 194.19	*D*_x_ = 1.394 Mg m^−^^3^
Monoclinic, *P*2_1_/*c*	Mo *K*α radiation λ = 0.71073 Å
Hall symbol: -P 2ybc	Cell parameters from 1628 reflections
*a* = 8.1943 (8) Å	θ = 2.0–25.0º
*b* = 12.0512 (11) Å	µ = 0.11 mm^−^^1^
*c* = 10.1067 (9) Å	*T* = 123 (2) K
β = 111.970 (3)º	Block, colourless
*V* = 925.57 (15) Å^3^	0.31 × 0.28 × 0.24 mm
*Z* = 4	

### Data collection

**Table:**

Bruker SMART CCD area-detector diffractometer	1623 independent reflections
Radiation source: fine-focus sealed tube	1487 reflections with *I* > 2σ(*I*)
Monochromator: graphite	*R*_int_ = 0.021
*T* = 123(2) K	θ_max_ = 25.0º
φ and ω scans	θ_min_ = 2.7º
Absorption correction: multi-scan(SADABS; Bruker, 2002)	*h* = −9→9
*T*_min_ = 0.969, *T*_max_ = 0.978	*k* = −13→14
9552 measured reflections	*l* = −11→12

### Refinement

**Table:**

Refinement on *F*^2^	Hydrogen site location: inferred from neighbouring sites
Least-squares matrix: full	H-atom parameters constrained
*R*[*F*^2^ > 2σ(*F*^2^)] = 0.035	*w* = 1/[σ^2^(*F*_o_^2^) + (0.0871*P*)^2^ + 0.1838*P*] where *P* = (*F*_o_^2^ + 2*F*_c_^2^)/3
*wR*(*F*^2^) = 0.115	(Δ/σ)_max_ < 0.001
*S* = 0.95	Δρ_max_ = 0.24 e Å^−^^3^
1623 reflections	Δρ_min_ = −0.19 e Å^−^^3^
128 parameters	Extinction correction: SHELXL97 (Sheldrick, 2008), Fc^*^=kFc[1+0.001xFc^2^λ^3^/sin(2θ)]^-1/4^
Primary atom site location: structure-invariant direct methods	Extinction coefficient: 0.288 (19)
Secondary atom site location: difference Fourier map	

### Special details

**Table:**

Geometry. All e.s.d.'s (except the e.s.d. in the dihedral angle between two l.s. planes) are estimated using the full covariance matrix. The cell e.s.d.'s are taken into account individually in the estimation of e.s.d.'s in distances, angles and torsion angles; correlations between e.s.d.'s in cell parameters are only used when they are defined by crystal symmetry. An approximate (isotropic) treatment of cell e.s.d.'s is used for estimating e.s.d.'s involving l.s. planes.
Refinement. Refinement of *F*^2^ against ALL reflections. The weighted *R*-factor *wR* and goodness of fit *S* are based on *F*^2^, conventional *R*-factors *R* are based on *F*, with *F* set to zero for negative *F*^2^. The threshold expression of *F*^2^ > σ(*F*^2^) is used only for calculating *R*-factors(gt) *etc*. and is not relevant to the choice of reflections for refinement. *R*-factors based on *F*^2^ are statistically about twice as large as those based on *F*, and *R*- factors based on ALL data will be even larger.

### Fractional atomic coordinates and isotropic or equivalent isotropic displacement parameters (Å^2^)

**Table:**

	*x*	*y*	*z*	*U*_iso_*/*U*_eq_	
O1	0.38082 (14)	0.30936 (8)	0.37656 (11)	0.0529 (3)	
H1	0.3162	0.3134	0.2897	0.079*	
O2	0.97787 (14)	−0.29441 (9)	0.29627 (10)	0.0516 (3)	
O3	1.11604 (14)	−0.37383 (8)	0.51277 (11)	0.0520 (3)	
N2	0.94810 (15)	−0.22777 (9)	0.49593 (12)	0.0440 (3)	
H2A	0.9787	−0.2353	0.5887	0.053*	
N1	0.83415 (14)	−0.14299 (9)	0.42392 (11)	0.0395 (3)	
C5	0.57987 (17)	0.04812 (11)	0.32350 (13)	0.0396 (4)	
H5	0.5728	−0.0024	0.2494	0.047*	
C6	0.69854 (16)	0.02765 (10)	0.46270 (13)	0.0369 (3)	
C3	0.47278 (17)	0.14106 (11)	0.29260 (13)	0.0408 (4)	
H3	0.3932	0.1538	0.1976	0.049*	
C1	0.48094 (16)	0.21605 (10)	0.39995 (14)	0.0390 (4)	
C2	0.59680 (18)	0.19579 (11)	0.53868 (14)	0.0428 (4)	
H2	0.6028	0.2459	0.6129	0.051*	
C4	0.70306 (17)	0.10317 (12)	0.56872 (14)	0.0410 (4)	
H4	0.7815	0.0904	0.6641	0.049*	
C8	1.01137 (16)	−0.29807 (11)	0.42353 (14)	0.0386 (4)	
C7	0.81502 (16)	−0.06805 (11)	0.50677 (14)	0.0398 (4)	
H7	0.8828	−0.0755	0.6058	0.048*	
C9	1.1945 (2)	−0.45473 (13)	0.4509 (2)	0.0619 (5)	
H9A	1.2675	−0.5054	0.5253	0.093*	
H9B	1.2678	−0.4171	0.4071	0.093*	
H9C	1.1018	−0.4969	0.3778	0.093*	

### Atomic displacement parameters (Å^2^)

**Table:**

	*U*^11^	*U*^22^	*U*^33^	*U*^12^	*U*^13^	*U*^23^
O1	0.0548 (6)	0.0432 (6)	0.0517 (6)	0.0084 (4)	0.0096 (5)	−0.0039 (4)
O2	0.0574 (6)	0.0589 (7)	0.0365 (6)	0.0024 (5)	0.0154 (5)	−0.0063 (4)
O3	0.0570 (6)	0.0504 (6)	0.0506 (6)	0.0140 (5)	0.0226 (5)	0.0073 (4)
N2	0.0520 (7)	0.0473 (7)	0.0344 (6)	0.0110 (5)	0.0181 (5)	0.0068 (5)
N1	0.0390 (6)	0.0411 (6)	0.0388 (6)	0.0018 (4)	0.0153 (5)	0.0040 (5)
C5	0.0446 (7)	0.0398 (7)	0.0358 (7)	−0.0025 (5)	0.0168 (5)	−0.0020 (5)
C6	0.0376 (6)	0.0367 (7)	0.0384 (7)	−0.0047 (5)	0.0164 (5)	0.0018 (5)
C3	0.0416 (7)	0.0423 (7)	0.0357 (7)	−0.0026 (5)	0.0111 (5)	0.0022 (5)
C1	0.0383 (7)	0.0340 (7)	0.0451 (8)	−0.0036 (5)	0.0161 (6)	0.0005 (5)
C2	0.0464 (7)	0.0408 (7)	0.0405 (7)	−0.0039 (6)	0.0155 (6)	−0.0063 (6)
C4	0.0421 (7)	0.0435 (8)	0.0352 (6)	−0.0041 (5)	0.0118 (5)	−0.0001 (5)
C8	0.0372 (7)	0.0407 (7)	0.0381 (7)	−0.0041 (5)	0.0141 (5)	−0.0006 (5)
C7	0.0419 (7)	0.0423 (8)	0.0351 (7)	−0.0020 (5)	0.0142 (5)	0.0020 (5)
C9	0.0611 (10)	0.0503 (9)	0.0785 (12)	0.0113 (7)	0.0311 (8)	−0.0005 (8)

### Geometric parameters (Å, °)

**Table:**

O1—C1	1.3595 (16)	C6—C4	1.3958 (18)
O1—H1	0.8400	C6—C7	1.4565 (18)
O2—C8	1.2114 (17)	C3—C1	1.3943 (19)
O3—C8	1.3431 (16)	C3—H3	0.9500
O3—C9	1.4339 (18)	C1—C2	1.3895 (19)
N1—N2	1.3917 (15)	C2—C4	1.3778 (19)
N2—H2A	0.8800	C2—H2	0.9500
N1—C7	1.2805 (17)	C4—H4	0.9500
N2—C8	1.3438 (17)	C7—H7	0.9500
C5—C3	1.3846 (18)	C9—H9A	0.9800
C5—C6	1.4007 (18)	C9—H9B	0.9800
C5—H5	0.9500	C9—H9C	0.9800
			
C1—O1—H1	109.5	C4—C2—C1	120.11 (12)
C8—O3—C9	116.56 (12)	C4—C2—H2	119.9
N1—N2—C8	119.88 (11)	C1—C2—H2	119.9
C8—N2—H2A	120.1	C2—C4—C6	121.73 (12)
N1—N2—H2A	120.1	C2—C4—H4	119.1
N2—N1—C7	113.47 (11)	C6—C4—H4	119.1
C3—C5—C6	120.82 (12)	O2—C8—O3	124.88 (12)
C3—C5—H5	119.6	O2—C8—N2	125.09 (13)
C6—C5—H5	119.6	O3—C8—N2	110.03 (11)
C4—C6—C5	117.73 (12)	N1—C7—C6	125.81 (11)
C4—C6—C7	117.09 (11)	N1—C7—H7	117.1
C5—C6—C7	125.16 (12)	C6—C7—H7	117.1
C5—C3—C1	120.46 (12)	O3—C9—H9A	109.5
C5—C3—H3	119.8	O3—C9—H9B	109.5
C1—C3—H3	119.8	H9A—C9—H9B	109.5
O1—C1—C2	117.44 (12)	O3—C9—H9C	109.5
O1—C1—C3	123.41 (12)	H9A—C9—H9C	109.5
C2—C1—C3	119.14 (12)	H9B—C9—H9C	109.5
			
C8—N2—N1—C7	−165.18 (12)	C5—C6—C4—C2	−0.82 (19)
C3—C5—C6—C4	0.84 (18)	C7—C6—C4—C2	−179.27 (11)
C3—C5—C6—C7	179.14 (12)	C9—O3—C8—O2	0.9 (2)
C6—C5—C3—C1	−0.14 (19)	C9—O3—C8—N2	−179.72 (12)
C5—C3—C1—O1	179.33 (12)	N1—N2—C8—O2	0.3 (2)
C5—C3—C1—C2	−0.60 (19)	N1—N2—C8—O3	−179.11 (10)
O1—C1—C2—C4	−179.31 (12)	N2—N1—C7—C6	−177.08 (11)
C3—C1—C2—C4	0.62 (19)	C4—C6—C7—N1	−176.85 (12)
C1—C2—C4—C6	0.1 (2)	C5—C6—C7—N1	4.8 (2)

### Hydrogen-bond geometry (Å, °)

**Table:**

*D*—H···*A*	*D*—H	H···*A*	*D*···*A*	*D*—H···*A*
O1—H1···O2^i^	0.84	2.58	3.068 (2)	118
O1—H1···N1^i^	0.84	2.11	2.941 (2)	169
N2—H2A···O2^ii^	0.88	2.13	2.964 (2)	158
C7—H7···O2^ii^	0.95	2.38	3.188 (2)	143

Symmetry codes: (i) −*x*+1, *y*+1/2, −*z*+1/2; (ii) *x*, −*y*−1/2, *z*+1/2.
